# **Predictors associated with low-risk women’s pre-labour intention for intrapartum pain relief: a cross-sectional study**

**DOI:** 10.1016/j.ijnsa.2022.100070

**Published:** 2022-02-19

**Authors:** Prof. Yvonne J Kuipers, Elise van Beeck

**Affiliations:** a Rotterdam University of Applied Sciences, Institute of Healthcare, Rochussenstraat 198, 3015 EK Rotterdam, Netherlands; b Edinburgh Napier University, School of Health and Social Care, 9 Sighthill Court, Edinburgh EH11 4BN, Scotland, UK

**Keywords:** Antenatal, Childbirth, Intention, Labour, Maternity services, Pain relief

## Abstract

**Background:**

Pregnant women have preferences about how they intend to manage labour pain. Unmet intentions can result in negative emotions and/or birth experiences.

**Objective:**

To examine the antenatal level of intention for intrapartum pain relief and the factors that might predict this intention.

**Design:**

A cross-sectional online survey-based study.

**Setting and participants:**

414 healthy pregnant women in the Netherlands, predominantly receiving antenatal care from the community-based midwife who were recruited via maternity healthcare professionals and social media platforms.

**Methods:**

The attitude towards intrapartum pain relief was measured with the Labour Pain Relief Attitude Questionnaire for pregnant women. Personality traits with the HEXACO-60 questionnaire, general psychological health with the Mental Health Inventory-5 and labour and birth anxiety with the Tilburg Pregnancy Distress Scale. Multiple linear regression was performed with the intention for pain relief as the dependant variable.

**Results:**

The obstetrician as birth companion (*p*<.001), the perception that because of the impact of pregnancy on the woman's body, using pain relief during labour is self-evident (*p*<.001), feeling convinced that pain relief contributes to self-confidence during labour (*p*=.023), and fear of the forthcoming birth (*p*=.003) predicted women were more likely to use pain relief. The midwife as birth companion (*p*=.047) and considering the partner in requesting pain relief (*p*=.045) predicted women were less likely to use pain relief.

**Conclusion:**

Understanding the reasons predicting women's intention of pain management during labour, provides insight in low-risk women's supportive needs prior to labour and are worth paying attention to during the antenatal period.

## 1 Contribution of the paper

What is already known about the topic?

• Nowadays, intrapartum pharmacological pain relief is common
• Discrepancy between pre-labour intention and actual intrapartum pain management can contribute to negative birth experiences

## 2 What this paper adds

• This study provides information to contribute to the action-intention gap between women's antenatal intention for and actual uptake of intrapartum pain relief
• The maternity care professional whom the woman expects to support her during labour, the woman's attitude regarding the impact of pregnancy and her self-confidence in coping with pain during labour predict the woman's intention for intrapartum pain relief
• The woman's partner plays an insignificant role in the woman's intention for intrapartum pain management

## 3 Introduction

Most women experience pain during labour. The World Health Organization regards pain management as a standard of quality of care, highlighting those maternity care professionals should address this timely, appropriately, and with respect for the woman's choice and needs. (WHO 2018) During the antenatal period, women are to be informed and encouraged to think about and to discuss their intrapartum pain relief expectations and preferences with their care provider, usually at the end of pregnancy. (Lally et al., 2014) The uptake of any pharmacological intrapartum pain relief amongst childbearing women worldwide, varies from 50 to 92% in primiparous women and from 12 to 68% in parous women, with variations between countries. (Seijmonsbergen-Schermers et al., 2020)

Women's intentions for intrapartum pain management and pain relief are associated with a multitude of reasons. Wanting to be in control of the pain or pain management, preventing to losing control or regaining control during labour influence women's intentions for wanting or not wanting pain relief. (Lally et al., 2014) Birth-related anxiety, (Adams et al., 2012; Koelewijn et al., 2017; Swift et al., 2017; Logtenberg et al., 2018) as well as personal background factors (e.g., age, ethnicity), parity (e.g. nulliparous or parous), choice of place of birth (e.g. hospital, home) and women's coping skills (e.g. catastrophising) (Schaal et al., 2020; Escott et al., 2009; Veringa et al., 2011; Klomp et al., 2013; Soliday et al., 2013; Wassen et al., 2013; Lindholm, 2015; Whitburn et al., 2019; Reitsma et al., 2020) are known to be of influence on the uptake or decline of intrapartum pain relief. Additionally, the woman's knowledge about pain management methods, her attitude towards labour and her perception of labour and labour pain, the actual course of labour, caregiver support and expected relief of the various pain management methods are associated with wanting or not wanting pain relief. (Lally et al., 2014; Whitburn et al., 2019; Low, 2002; Heinze and Sleigh, 2003; Van den Bussche et al., 2007; Williams et al., 2008; Christiaens et al., 2010; Olza et al., 2018; Thomson et al., 2019; Newnham et al., 2021)

Nowadays, intrapartum pharmacological pain relief is becoming much more common, as well as the woman's right to choose a pain-free birth is regarded as a legitimate feminist position. (Lindholm, 2015; Skowronski, 2015) The fact that intrapartum pain relief is nowadays available and accessible might influence the normalisation of pain relief. (Whitburn et al., 2019; Stoll et al., 2014) At the same time, labour and birth are currently more medicalised and intervened, being associated with the uptake of pharmacological pain relief, often as part of a cascade of interventions. (Whitburn et al., 2019; Rossignol et al., 2014; Whitburn et al., 2014) Midwives experience a change in attitude to labour pain amongst women - with an increasing tendency towards pain relief - and with the midwife's woman-centred focus, midwives tend to fulfil the women's wishes. (Lindholm, 2015) Pregnant women's intentions are also affected by social and medical influences, such as messages received via media and via health professionals but mostly through social (media) networks of family members and peers. (Lally et al., 2014; Lindholm, 2015; Thomson et al., 2019; Stoll et al., 2014; MacLean, 2014; Beeck van et al., 2019; Miller and Danoy-Monet, 2021) The partners’ opinion about intrapartum pain relief influences women's uptake of pain relief, (Orbach-Zinger et al., 2008; Harkins et al., 2010) especially when partners feel helpless or not included in the care process before and/or during labour. (Bäckström and Hertfelt Wahn, 2011) Moreover, the attitude of maternity care professionals towards intrapartum pain (relief) and organisational, environmental and contextual barriers in maternity services (e.g. availability, resources, continuity of caregiver) are factors that influence women's intentions for intrapartum pain relief. (Christiaens et al., 2010; McCauley et al., 2018)

A discrepancy between pre-labour intention and actual intrapartum pain management has been reported. (Lally et al., 2008) When women's intrapartum pain relief wishes are not met -either administration of pharmacological pain relief without having the intention, not receiving pain relief while intending to do so, or other type pain management than preferred- can contribute to negative birth experiences. (Orbach-Zinger et al., 2008; Webb et al., 2021) Negative experiences are associated with an increased risk to develop postpartum depression or even postpartum post-traumatic stress disorder. (Thomson et al., 2019; Orbach-Zinger et al., 2008; Webb et al., 2021) In comparison to women who give birth without pharmacological pain relief, women who use pharmacological pain relief, without intending to do so, more often express negative feelings such as guilt or failure about the unexpected need for pharmacological pain relief. (Thomson et al., 2019) Aligning women's intention of using pharmacological pain relief with the actual uptake during labour, contributes to women's positive birth experience. (Karlsdottir et al., 2018) The degree to which pain relief management diverges from the imagined and idealised birth, could affect women's long-term psychosocial wellbeing. (Larkin et al., 2009) Discrepancies between expectations and experiences have been shown to be critical in birth satisfaction and childbirth narratives. (Soliday et al., 2013; Webb et al., 2021; Koster et al., 2019) This suggests that intention and actual intrapartum pain management should ideally be congruent.

In the Netherlands, there is a 45.3% pharmacological pain relief rate amongst women with singleton term childbirths from 37 weeks of gestation onwards, (Perined 2021) of which 22.8% epidural analgesia, with variations between care providers (midwife-led care 8–29%, obstetrician-led care 18–53%). (Perined 2021; Seijmonsbergen-Schermers et al., 2018) Pharmacological pain relief options consist of pethidine injections, intravenous patient-controlled Remifentanil administration, and some hospitals offer nitrous oxide as a form of pain relief. Dutch data on pharmacological pain relief and non-pharmacological pain relief such as intracutaneous sterile water injections, transcutaneous electrical nerve stimulation (TENS), acupuncture, reflexology, breathing and relaxation techniques, and water immersion are unavailable. (Klomp et al., 2013; Perined 2021; FMS 2020) Worthy of note is that in the Netherlands intrapartum pain relief, apart from nitrous oxide, can only be administered under the auspices of the obstetrician. When women are in the care of the midwife at the start of labour and need pain relief, the midwife is no longer involved in the woman's intrapartum care and refers the woman to the obstetrician as lead caregiver, handing over the responsibility of care. (Klomp et al., 2013) About 15% of labouring women in the care of midwives are being referred for epidural analgesia and approximately a third of these women's midwives stay with the woman, despite they are no longer the primary care giver. (Perined 2021; Fontein, 2010) Dutch registration shows that the highest uptake for epidural (58%) is amongst women who have an induction of labour. (Perined 2021)

Women value when being encouraged and supported by maternity care professionals in their intentions either to labour with or without pain relief. (Leap et al., 2010) Most professionals are open to helping women in their pain management choices. (McCauley et al., 2018) We hypothesize that when we have more knowledge about the determinants associated with women's antenatal intentions for pain relief during labour, we are better able to support women during pregnancy in encouraging and empowering them, to achieve their intended method of pain management and to provide support that will help them to pursue their antenatal intention during labour - varying from not wanting pain relief to wanting pain relief. When maternity care professionals are prepared to meet women's supportive needs prior to labour, they might be better able to adequately support women in preventing the pain management action-intention gap - aiming to prevent women's potential negative feelings and emotions and negative birth experiences resulting from the discrepancy between intention and actual intrapartum pain management.

In this study, we wanted to examine the levels of intention to request intrapartum pain relief in a population of Dutch pregnant women, to identify the explicit factors that might serve as a proxy for maternity service providers to support women during antenatal care in pursuing their intention for pain relief management during birth. To fulfil this purpose, we sought an answer to the following question: Which factors are associated with the intention of pregnant women to giving birth with or without pain relief? The paper is of cross-cultural interest as intrapartum pain management is a phenomenon occurring in Mondial maternity care.

## 4 Methods

The data were collected between 21 February and 19 September 2019, using online self- completed questionnaires (Limesurvey©). The participants were recruited by combining convenience and purposive sampling. Healthcare professionals in primary and secondary care settings (midwives, obstetricians, doula's, general practitioners, antenatal educators) were informed about the study and asked to spread flyers and posters announcing the study and inviting potential participants. The posters and flyers included a hyperlink and Quick Response-code, anonymously redirecting the participant to the questionnaire. Participants were also recruited via social media platforms (Twitter©, Instagram©, Facebook©), allowing snowballing. We included women who were 18 years of age or older and during any trimester of pregnancy, receiving care from a community midwife or obstetrician. The community midwife in the Netherlands has a caseload of healthy women with uncomplicated pregnancies (low risk), the obstetrician's holds a caseload of women with (medically) complicated pregnant women (high risk). Approximately 90% of all women start their antenatal care with the midwife, 45% are being referred to the obstetrician during pregnancy and again half of these women are being referred during labour. (Perined 2021)

### 4.1 Measures

The questionnaire included socio-demographic and personal details (e.g., age, gestation, parity, relation, job). Participants were asked if they: (1) did not intend to use intrapartum pain relief; (2) had not made a decision yet about pain relief; (3) would maybe use some form of pain relief at some point during labour; (4) would probably opt for an epidural, but no other form of pain relief; (5) did intend/were definite about any form of pain relief during labour but most likely an epidural from the start of labour/early labour. Questions could be answered with ‘yes’ or ‘no’. We asked the participants to indicate from which person they expected to be (emotionally) supported during labour and birth (i.e., labour & birth companionship). The survey included the Dutch versions of the validated self-report measures: HEXACO-60, Tilburg Pregnancy Distress Scale (TPDS), Mental Health Inventory (MHI-5) and Labour Pain Relief Attitude Questionnaire for pregnant women (LPRAQ-p). (De Vries et al., 2009; CBS 2011; VJ Pop et al., 2011; Hulsbosch et al., 2020)

### 4.2 HEXACO-60

The six-factor HEXACO model of personality emerged as an alternative to the Five-Factor Model. (Lee and Ashton, 2004) The HEXACO-60 questionnaire assesses six personality dimensions: Honesty-Humility, also known as integrity (H), Emotionality (E), Extraversion (X), Agreeableness (A), Conscientiousness (C), and Openness to Experience (O) via agreement on a five-point scale (1 = strongly disagree, 5 = strongly agree) to ten self-descriptive statements per trait. Honesty-Humility (integrity) includes sincerity, fairness, modesty, and greed-avoidance. Emotionality includes fearfulness, anxiety, dependence, and sentimentality and Extraversion includes social self-esteem, social boldness, sociability, and liveliness. Agreeableness includes forgiveness, gentleness, flexibility, and patience. Conscientiousness organisation, diligence, perfectionism, and prudence. Openness includes aesthetic appreciation, inquisitiveness, creativity, and unconventionality. (Ashton and Lee, 2009) The HEXACO domains have shown good internal consistency (α = 0.71–0.79). (Bartley and Roesch, 2011)

### 4.3 Mental health inventory (MHI-5)

The Mental Health Inventory (MHI-5) is the mental health subscale of the 36-item Short-Form Health Survey Questionnaire (SF-36). (Ware and Sharebourne, 1992) The MHI-5 is a brief, valid and reliable tool for detecting psychological wellbeing in the general population, (Ware et al., 2000; Cuijpers et al., 2009) showing good internal consistency (α.= 85) in a Dutch-speaking population. (Dijkstra et al., 2005; Dykstra et al., 2012; Hogerbrugge et al., 2014) The MHI-5 consists of the following five questions: How much of the time during the last four weeks, have you…: (i) ‘Been a very nervous person?’ (ii) ‘Felt so down in the dumps that nothing could cheer you up?’, (iii) ‘Felt calm and peaceful?’ (iv) ‘Felt downhearted and blue?’, (v) ’Been a happy person?’. Each item has six possible responses ranging from ‘all the time’ (0) to ‘at no time’ (5). After computing the MHI-5 values (multiplying each score by four) the total score varies from 0 to 100, with higher scores indicating good mental health. (Driessen, 2011) Although within the NKPS no MHI-5 cut-off point was utilized, based on a Dutch general population-base study, an MHI-5 score of ≤60 was regarded as the cut-off value as indicators of cases with reduced psychological wellbeing. (Perenboom et al., 2000; Hoeymans et al., 2004) This cut-off point is a recognised value to minimize misclassification in a study aiming to identifying cases in a specific context (i.e., pregnancy). (Kelly et al., 2008)

### 4.4 Tilburg pregnancy distress scale (TPDS)

The TPDS consists of 16 items and two subscales as it explores the negative affect (TPDS-NA) (11 items) related to the woman's pregnancy and birth and it explores the woman's perception of partner involvement (5 items TPDS-PI). The TPDS-NA includes five specific birth-related items. The TPDS uses a 4-point rating scale (0 = very often, to 3 = rarely or never) generating a total score ranging from 0 to 48. A total TPDS 16-item score above 17 indicates an increased negative affect towards pregnancy and birth. A total TPDS-NA score above 12 indicates birth and health-related anxiety and fear. The TPDS-NA contains 5 items about fear towards the forthcoming birth/confinement (TPDS-C). (VJM Pop et al., 2011) In the Dutch validation studies amongst pregnant women, the TPDS showed good psychometric properties and good internal consistency (α.= 78, α.80) for the 16-item scale and acceptable internal consistency (α.71) for the 11-items TPDS-NA scale. (VJM Pop et al., 2011; Boekhorst et al., 2019; Kuipers et al., 2020) The TPDS was developed and validated amongst Dutch women and practitioners to create a scale primarily reflecting pregnant women's experiences of birth-related anxiety and fear. (VJM Pop et al., 2011) Although the TPDS is a Dutch developed scale tested within a Dutch childbearing population, the scale is also being used internationally. (Boekhorst et al., 2019)

### 4.5 Labour pain relief attitude questionnaire for pregnant women (LPRAQ-p)

The LPRAQ-p consists of 6 items formulated as statements, using a five-point score ranging from ‘totally disagree’ (1) to ‘totally agree’ (5). A higher score showing higher level of agreement with the statement. The questionnaire was developed after focus group discussions and was validated by 861 Dutch childbearing women, showing good psychometric properties: a two-factor structure with acceptable to good internal consistency (α.79 to 0.84) and excellent model fit (CFI = 0.99, NFI = 0.98, TLI = 0.99, RMSEA = 0.02, lower bound = 0.01). (Hulsbosch et al., 2020)

## 5 Analysis

An *a priori* sample size calculation, with statistical significance set at *p* 0.05 (95% CI), showed we required a minimum of 378 participants in this study. Normality of distribution was checked using visual interpretation of histograms and QQ-plots. When >10% of the values per case were missing, the case was deleted and in case of <10% missing values, the values were imputed with the mean score. We calculated descriptive statistics for the sociodemographic and personal characteristics. Cronbach's alpha (α) was calculated to measure internal consistency of the HEXACO categories, MHI-5 and the TPDS. Sum scores were calculated for the six HEXACO domains, MHI-5, TPDS 16 items, TPDS-NA 11 items, TPDS-PI 5 items and for the TPDS-C 5 items. (VJM Pop et al., 2011) The strategy for model building was as follows: a continuous variable (intention pain relief) was constructed to be used as the dependant variable in the multiple linear regression analysis. We recoded the positive answers (yes) into new variables: ‘No intention for pain relief was recoded’ in 1, ‘undecided about any form of pain relief’ in 2; ‘maybe pain relief at some point during labour’ in 3; ‘probably an epidural right from the start of labour/ early labour but no other form of pain relief’ in 4; ‘definite about any form of pain relief during labour but most likely an epidural from the start of labour/early labour’ was recoded in 5. The lower the score, the lower the intention to use pain relief during labour (from no intention to intention, a minimum-maximum score of 1–5). One-sample *t*-test (continuous data) and Chi-square (dichotomous data) were used to calculate differences between responders and non-responders. One-way ANOVA, Kruskal-Wallis test and Chi-square were used to calculate differences between background characteristics and intention for pain relief. ANOVA with post hoc Bonferroni correction assessed the ordinal variable variances to decide on dummy variables. Multiple linear regression analysis was used to examine the relationship between the dependant variable (intention pain relief) and the multiple independent variables. The correlation matrix was checked for multicollinearity of the independent variables. The analyses were performed using the Statistical Package for the Social Sciences© (SPSS) version 26.

## 6 Ethical approval

The study protocol was reviewed and approved by the Antwerp University Hospital Ethics Committee as part of Dutch/Flemish research project (Reference nr. B300201942200). According to Dutch legislation, additional ethical approval was not necessary. (CCMO 2002) The study was performed in accordance with the ethical standards of the 1964 Helsinki declaration and its later amendments. Participation was voluntary. Before consenting, the participant was directed to a webpage with information about the study. Informed consent was obtained via box ticking before the questionnaire could be completed.

## 7 Results

### 7.1 Participants

**Fig. 1:**
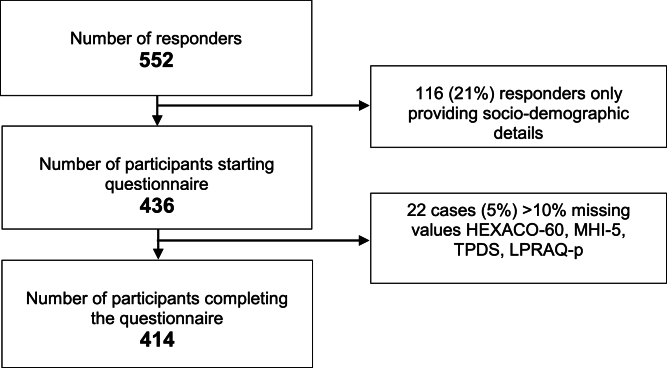
Flowchart.

Of the 552 responders, 414 participants completed the questionnaire (completion rate 75%) (Fig. 1). We observed no significant differences between the socio-demographic details of the participants who completed the questionnaire (*n* = 414) and who did not (*n* = 138). All participants met the inclusion criteria.

Most of the participants had a Dutch background, were in a relationship and had a job. Most participants were in the second and third trimester of pregnancy (83.1%) and were pregnant of a second or subsequent child (66%). Most of the participants received antenatal care from the community-based midwife (92.3%). Approximately 1 in 10 women of the sample showed reduced general and pregnancy/birth specific psychological wellbeing. Women appointed predominantly their partner or the midwife as their primary companion during labour and birth (92.9%) (Table 1).

**Table 1:** Socio-demographic and personal details participants.

	**All participants (*n*** **=** **414)**	
	**N (%)**	**Mean (SD±) range**
**Age in years**		29.77 (±4.2)19–45
**Ethnic background** born in the Netherlands not born in the Netherlands	400 (95.6) 14 (3.4)	
**Partnership** cohabiting not cohabiting	401 (96.8) 13 (3.2)	
**Highest level of education** elementary school (pre-vocational) secondary education higher secondary education higher education (bachelor & university)	2 (0.5) 36 (8.7) 108 (26.1) 268 (64.7)	
**Daily/job (activities)** paid job *full-time* *part-time* no (paid) job student	381 (92) *127 (33.3)* *254 (66.7)* 25 (6) 8 (2)	
HEXACO-60 (H)integrity (α.76) Emotionality (α.78) eXtraversion (α.79) Agreeableness (α.70) Conscientiousness (α.70) Openness to experience (α.77)		3.10 (±.30) 1–4 2.92 (±.32) 1–3.8 2.59 (±.30) 1–3.4 2.97 (±.33) 1–3.8 2.89 (±.31) 1–3.6 2.87 (±1) 1–4
**MHI-5^a^ (α.78)** total score below cut-off (≤60)	45 (10.9)	72.82 (±12.8) 0–100
**Gravidity**		2.30 (±1.4) 0–14
**History pregnancy loss**	107 (28.5)	
**Parity** nulliparous parous	141 (34) 273 (66)	0.89 (±.81) 0–5
**Gestation in weeks**		23.92 (±11.09) 0–41
**Trimester of pregnancy** first trimester (0–12 weeks) second trimester (13–26 weeks) third trimester (27–42 weeks)	70 (16.9) 160 (38.7) 185 (44.7)	
**Primary caregiver antenatal period** obstetrician community midwife shared antenatal care obstetrician & midwife	8 (1.9) 387 (93.5) 19 (4.6)	
**Labour & birth companion (assigned by woman)** midwife partner friend/family member doula obstetrician	212 (50) 167 (41.5) 9 (2.2) 6 (1.5) 20 (4.8)	
**TPDS^b^ total score TPDS 16-items (α.73)** above cut-off >17 total score TPDS-NA^c^ 11 items (α.79) above cut-off >12 total score TPDS-PI^d^ 5 items (α.83) total score TPDS-C^e^ 5 items (α.84)	52 (12.5) 38 (9.2)	12.32 (±5) 0–33 7.73 (±6.4) 0–33 4.77 (±2.7) 0–15 3.29 (±2.7) 0–15

^a^Mental Health Inventory 5 items

^b^Tilburg Pregnancy Distress Scale

^c^Tilburg Pregnancy Distress Scale-Negative Affect.

^d^Tilburg Pregnancy Distress Scale-Partner Involvement.

^e^Tilburg Pregnancy Distress Scale-Confinement.

## 8 Descriptives

Most women intended not to use pain relief during labour (73.4%), 23.4% was interested but undecided while only 3.2% of the sample was definite about wanting pain relief during birth. The mean scores of the PRAQ-p were low within the 1–5 scale range (Table 2). We observed differences between women with different pain relief intentions: gravidity (*p* < 0.001), parity (*p* < 0.001), gestational period (*p* < 0.001), trimester of pregnancy (*p* < 0.001), labour & birth companion (*p* = .001), all LPRAQ-p items (*p* < 0.001; *p* < 0.001; *p* < 0.001; *p* < 0.001; *p* = .01; *p* = .002), the HEXACO categories Emotionality (*p* <0.001), Conscientiousness (*p* = .01) and Openness to experience (*p* = .006), MHI-5 total score (*p* = .045), and TPDS-NA above cut off score (*p* < 0.001). ANOVA Bonferroni post hoc test (*F*(5.41) = 0.3.47, *p* < 0.001) showed differences in intention when the midwife was the assigned labour & birth companion (*p* = .006) as well as the obstetrician was the assigned labour & birth companion (*p* = .04). ANOVA Bonferroni post hoc test (*F*(6.53) = 4.41, *p* < 0.001) showed difference in intention for pain relief between the second and third trimester of pregnancy (*p* < 0.001; *p* < 0.001) (Table 3).

**Table 2:** Intention intrapartum pain relief and labour pain relief attitude.

	**All women (*n*** **=** **414)**	
	**N (%)**	**Mean (SD ±) range**
**Intention pain relief (any form)** No intention for pain relief Undecided about pain relief Maybe pain relief at some point during labour Probably EA* but no other form of pain relief Definite any form of pain relief during labour but most likely EA* right from the start of labour/ early labour	304 (73.4) 31 (7.5) 41 (9.9) 25 (6.0) 13 (3.2)	
**Labour Pain Relief Attitude^a^ (α.72)** 1.Because my pregnancy has already had a big impact on my body, I think it is normal to ask for pain relief 2.I also ask for pain relief because of my partner 3.I am convinced that if I get pain relief, I will feel more self-confident during labour 4. Pain relief will help me to perform better during labour 5.My partner plays an important role in the decision to ask for pain relief during labour 6.My friends and relatives play an important role in the decision to ask for pain relief during labour		2.14 (±.98) 1–5 1.25 (±.56) 1–4 2.01 (±.90) 1–5 2.06 (±.90) 1–5 1.93 (±.97) 1–5 1.31 (±.56) 1–4

^a^Measured with Labour Pain Relief Attitude Questionnaire for pregnant women (LPRAQ-p).

*Epidural Anaesthesia.

**Table 3:** Differences participants’ characteristics in relation to level of intention pain relief (*n* = 414).

**Participants’ characteristics**	Levels of intention pain relief				
	**1**^a^(*n* = 304)	**2**^a^(*n* = 31)	**3**^a^(*n* = 41)	**4**^a^(*n* = 25)	**5**^a^(*n* = 13)
	**Mean (SD±) range**				
**Age**	29.9 (±4.2) 19–45	29.283(±3.8) 22–42	30.71 (±3.7) 22–38	28.9 (±3.8) 22–34	27.7 (±3.9) 21–33
**HEXACO** (H)integrity Emotionality^⁎⁎⁎^ eXtraversion Agreeableness Conscientiousness^⁎⁎^ Openness to experience^⁎⁎^	3.1 (±.30) 1–4 2.9 (±.29) 1–3.7 2.6 (±.30) 1–3.4 3.0 (±.32) 1–3.6 2.9 (±.31) 1–3.6 2.8 (±.29) 1–3.6	3.0 (±.30) 2.2–3.4 2.7 (±.44) 2–3.4 2.6 (±.37) 2–3.2 3.0 (±.33) 2.3–3.5 2.8 (±.30) 2.2–3.2 2.7 (±.28) 2.2–3.3	3.0 (±.36) 2–3.8 3.1 (±.34) 2.5–3.8 2.6 (±.26) 1.9–3 3.0 (±.35) 2.2–3.5 3.0 (±.28) 2.4–3.4 2.8 (±.29) 2.2–3.3	3.2 (±.25) 2.9–3.6 2.8 (±.36) 2.4–3.6 2.6 (±.26) 1.9–3 3.0 (±.37) 2.4–3.5 2.9 (±41) 2.1–3.5 2.6 (±.59) 1.6–3.5	3.1 (±.20) 2.8–3.4 2.9 (±.45) 2.3–3.5 2.6 (±.36) 2.2–3.2 3.1 (±.34) 2.8–3.8 2.8 (±.28) 2.5–3.2 2.8 (±.31) 2.4–3.2
**Gravidity^⁎⁎⁎^**	2.41 (±1.5) 1–14	1.7 (±.66) 1–3	2.4 (±.97) 1–5	1.5 (±.71) 1–3	2.2 (±1.1) 1–4
**Parity^⁎⁎⁎^**	1 (±.81) 0–3	.5 (±.57) 0–2	1 (±.67) 0–3	.4 (±.49) 0–1	1 (±.82) 0–2
**Gestation^⁎⁎⁎^**	24.3 (±11.5) 0–41	20.3 (±11.2) 0–37	28.2 (±7.9) 10–40	20.8 (±8.3) 0–37	16.9 (±6.6) 6–27
**Labour Pain Relief Attitude (LPRAQ-p)^b^** LPRAQ-p 1^⁎⁎⁎^ LPRAQ-p 2^⁎⁎⁎^ LPRAQ-p 3^⁎⁎⁎^ LPRAQ-p 4^⁎⁎⁎^ LPRAQ-p 5^⁎⁎^ LPRAQ-p 6^⁎⁎^	1.8 (±.64) 1–4 1.2 (±.48) 1–4 1.8 (±.73) 1–4 1.8 (±.71) 1–4 1.8 (±.87) 1–4 1.3 (±.48) 1–4	3.4 (±.88) 1–5 1.9 (±.79) 1–4 2.5 (±.96) 1–4 2.6 (±.81) 1–4 2.5 (±1.3) 1–4 1.6 (±.68) 1–3	2.7 (±1.1) 1–4 1.3 (±.67) 1–3 2.2 (±.1.1) 1–4 2.4 (±1.1) 1–4 1.9 (±1.0) 1–4 1.5 (±.71) 1–4	3.3 (±.85) 2–4 1.3 (±.46) 1–2 3.0 (±.79) 2–4 3.0 (±1.0) 1–4 2.4 (±1.0) 1–4 1.5 (±.77) 1–3	3.7 (±1.1) 2–5 1.0 (±.0) 1 3.2 (±1.4) 1–5 3.2 (±1.3) 1–5 2.1 (±1.5) 1–5 1.4 (±.78) 1–3
MHI-5^c^ total score*	75.6 (±10.6) 20–96	73.3 (±12.4) 40–88	70.1 (±11.7) 40–88	76.2 (±10.9) 52–100	74.2 (±16.9) 40–92
**TPDS^d^ total score**	12.1 (±4.9) 3–33	13.4 (±5.5) 3–20	14.1 (±4.4) 4–26	11.0 (±5.3) 3–20	11.6 (±5.6) 8–23
**TPDS-NA^e^**	7.2 (±4.4) 0–33	7.9 (±5.2) 0–20	9.3 (±3.9) 2–16	7.1 (±3.5) 2–12	7.0 (±3.3) 2–13
**TPDS-PI^f^**	4.8 (±2.6) 0–14	4.7 (±3.3) 0–13	4.8 (±2.7) 0–11	3.9 (±2.5) 0–9	4.6 (±3.3) 1–10
**TPDS-C^g^**	3.1 (±2.6) 0–15	3.8 (±3.5) 0–13	4.2 (±1.7) 0–8	3.3 (±3.3) 0–9	3.9 (±2.9) 0–7
	**N (%)**				
**Ethnic background** born in the Netherlands not born in the Netherlands	294 (96.7) 10 (3.3)	31 (100) -	39 (95.1) 2 (4.9)	23 (92) 2 (8)	13 (100) -
**Partnership** cohabiting not cohabiting	292 (96) 12 (4)	30 (96.8) 1 (3.2)	41 (100) -	25 (100) -	13 (100) -
**Education** elementary school (pre-vocational) secondary education higher secondary education higher education & university)	2 (0.7) 105 (34.5) 3 (1) 194 (63.8)	- 12 (38.7) - 19 (61.3)	- 7 (17.1) 1 (2.4) 33 (80.5)	- 11 (44) - 14 (56)	- 5 (38.5) - 8 (61.5)
**Daily/job (activities)** paid job no (paid) job student	274 (90.1) 22 (7.2) 8 (2.7)	30 (96.8) 1 (3.2) -	41 (100) - -	25 (100) - -	11 (84.6) 2 (15.4) -
**History of pregnancy loss**	79 (26)	5 (16.1)	16 (39)	5 (20)	2 (15.4)
**Trimester of pregnancy^⁎⁎⁎^** first trimester (0–12 weeks) second trimester (13–26 weeks) third trimester (27–42 weeks)	55 (18.1) 104 (34.2) 145 (47.7)	7 (22.6) 14 (45.1) 10 (32.3)	3 (7.3) 13 (31.7) 25 (61)	2 (8) 20 (80) 3 (12)	3 (23.1) 8 (61.5) 2 (15.4)
**Caregiver antenatal period** obstetrician community midwife shared care	8 (2.6) 282 (92.8) 14 (4.6)	- 30 (96.8) 1 (3.2)	- 39 (95.1) 2 (4.9)	- 25 (110) -	- 11 (84.6) 2 (15.4)
**Labour & birth companion^⁎⁎⁎^** **midwife** partner friend/family member doula obstetrician	154 (50.7) 131 (43.1) 7 (2.3) 6 (2) 6 (1.9)	23 (74.2) 8 (25.8) - - -	21 (51.2) 18 (43.9) 2 (4.9) - -	12 (48) 9 (36) - - 4 (16)	2 (15.4) 1 (7.7) - - 10 (76.9)
**MHI-5^c^ below cut-off (≤60)**	29 (9.5)	6 (19.4)	6 (14.6)	2 (8)	2 (15.4)
**TPDS^d^ above cut-off (>17)**	34 (11.2)	8 (25.8)	6 (14.6)	2 (8)	2 (15.4)
**TPDS-NA^e^ above cut-off (>12)^⁎⁎⁎^**	19 (6.3)	5 (16.1)	12 (29.3)	–	2 (15.4)

^a^1. No intention for pain relief; 2. Undecided about any form of pain relief; 3. Maybe pain relief at some point during labour; 4. Probably an epidural right from the start of labour/early labour but no other form of pain relief; 5. Definite about any form of pain relief during labour, but most likely an epidural from the start of labour/early labour.

^b^1.Because my pregnancy has already had a big impact on my body, I think it is normal to ask for pain relief; 2.I also ask for pain relief because of my partner; 3.I am convinced that if I get pain relief, I will feel more self-confident during labour; 4. Pain relief will help me to perform better during labour; 5.My partner plays an important role in the decision to ask for pain relief during labour; 6.My friends and relatives play an important role in the decision to ask for pain relief during labour.

^c^Mental Health Inventory 5 items.

^d^Tilburg Pregnancy Distress Scale.

^e^Tilburg Pregnancy Distress Scale-Negative Affect.

^f^Tilburg Pregnancy Distress Scale-Partner Involvement.

^g^Tilburg Pregnancy Distress Scale-Confinement.

*p < .05 (2-tailed).

^⁎⁎^*p* ≤ .01 (2-tailed).

^⁎⁎⁎^*p* ≤ .001 (2-tailed).

## 9 Multiple linear regression analysis

Gravidity and parity, gestational period, and trimesters of pregnancy, and TPDS total score and TPDS-C showed multicollinearity (*r* = 0.80; *r* = 81, *r* = 0.89). We entered gestational period, parity, midwife and obstetrician as labour & birth companion, the labour pain relief items, the HEXACO categories Emotionality, Conscientiousness and Openness to experience, MHI-5 total score, and TPDS-C score as independent variables. We chose TPDS-C 5 as this is the labour-specific component of the TPDS. (Lamm et al., 2011) Intention for pain relief was entered as the dependant variable. Multiple linear regression analysis shows that the obstetrician as appointed labour & birth companion (*p* < 0.001), the perceptions: that pregnancy has already had a big impact on the woman's body, it is normal to ask for pain relief (*p* < 0.001), feeling convinced that pain relief contributes to self-confidence during labour (*p* = .023), and fear of the forthcoming birth (*p* = .003) have a significant positive relationship with the intention for pain relief. The midwife as labour & birth companion (*p* = .047) and requesting pain relief because of the woman's partner (*p* = .016), have a significant negative relationship with the intention for pain relief. The amount of variance in the independent variable accounted for by the independent variables was 38% (Table 4).

**Table 4:** Multiple linear regression analysis of predictors of intention for pain relief during labour (*n* = 414).

	95% Confidence Interval (CI) for B						
	B	SE B	ß	t	Sig (p)	Lower bound	Upper bound
**(Constant)**	−0.926	.762		−1.262	.208	−2.370	.517
**Length gestational period**	.002	.004	.018	.448	.654	−0.006	.010
**Parity (number of births)**	−0.021	.058	−0.015	−0.362	.718	−0.135	.093
Midwife as labour & birth companion (vs partner & obstetrician)	**−0.206**	**.089**	**.095**	**−2.305**	.022*	**−0.381**	**−0.030**
Obstetrician as labour & birth companion (vs partner & midwife)	**.500**	**.246**	**.083**	**2.034**	.043*	**.584**	**2.210**
Because my pregnancy has already had a big impact on my body, I think it is normal to ask for pain relief	**.576**	**.060**	**.517**	**9.546**	**<0.001^⁎⁎⁎^**	**.458**	**.695**
I also ask for pain relief because of my partner	**−0.204**	**.084**	**−0.105**	**−2.422**	.016*	**−0.370**	**−0.038**
I am convinced that if I get pain relief, I will feel more self-confident during labour	**.172**	**.075**	**.142**	**2.276**	.023*	**.023**	**.320**
**Pain relief will help me to perform better during labour**	.039	.082	.032	.472	.637	−0.123	.200
**My partner plays an important role in the decision to ask for pain relief during labour**	−0.072	.050	−0.064	−1.421	.156	−0.171	.027
**My friends and relatives play an important role in the decision to ask for pain relief during labour**	.075	.085	.039	.889	.375	−0.091	.241
**Emotionality**	.145	.144	.043	1.003	.317	−0.139	.429
**Conscientiousness**	.026	.149	.008	.175	.861	−0.266	.318
**Openness to experience**	−0.126	.146	−0.037	−0.859	.391	−0.414	.162
**MHI-5 (mental health)**	.001	.004	.003	.069	.945	−0.008	.009
TPDS-C	**.109**	**.036**	**.247**	**3.018**	**.003^⁎⁎^**	**.038**	**.181**

F 16.030. Model significance <0.001. R 0.61. Nagelkerke R^2^.38. Adjusted Nagelkerke R^2^.36.

*p < .05 (2-tailed).

^⁎⁎^*p* ≤ .01 (2-tailed).

^⁎⁎⁎^*p* ≤ .001 (2-tailed).

## 10 Discussion

We aimed to examine the level of intention for intrapartum pain relief of Dutch pregnant

women and to identify the associated predictors. Knowing that the uptake of pharmacological pain relief amongst Dutch childbearing women is 45.3%, (Perined 2021) the discrepancy between the intention of the women in our study – with 73.4% of women not wanting pain relief during labour - and the actual national registered administration is evident. This emphasizes the need for preventing the pain management action-intention gap, indicating that the woman's labour companion, the woman's attitude towards pain relief, and the role of the woman's partner are aspects worth paying attention to during the antenatal period to support women in pursuing their goals.

Our regression model shows that either assigning the midwife or the obstetrician as labour and birth companion is associated with women's intention for pain relief. This is congruent with an earlier Dutch study, reporting that a woman's preference for not wanting pain relief is more likely amongst women who receive midwife-led care compared to obstetric-led care. (Wassen et al., 2013) As in the Dutch maternity system, the obstetrician is the only professional to provide pain relief, it might be logical that high intentions are associated with the obstetrician as caregiver - as the meaning of pain and the meaning of the context where pain exists affects making sense of the pain, it predicts intrapartum pain experiences and choices how to cope with pain. (Whitburn et al., 2019; Whitburn et al., 2014; Chapman et al., 2008; Klein et al., 2009; Aune et al., 2021) The woman's intention can thus simply be a response to the care professional's scope of practice and their possibilities of pain management. (Lamm et al., 2011; Coulm et al., 2015) Assigning the obstetrician -a medically orientated professional- midwife -a more physiologically orientated professional- might illustrate the meaning of labour pain. (Whitburn et al., 2019) Giving meaning and making sense of labour pain is very much in line with the salutogenic approach which emphasizes the manageability (understanding the pain, coping with pain), the meaningfulness (assigning meaning to the pain) and comprehensibility (fears and emotions) when discussing intrapartum pain management with women during the antenatal period. (Perez-Botella et al., 2015) Discussing these issues require in-depth conversations between the woman and her caregiver, which might be more beneficial to women rather than restricting the conversation by informing women about risks and benefits of pain relief and asking them at the end of pregnancy to make decisions about intrapartum pain relief. (Lally et al., 2014; Lindholm, 2015) Discussing the topic earlier during pregnancy is enhanced by our regression model, which did not show a significant association between intention for intrapartum pain relief and gestational period. This suggests that women might have ideas about their needs and wishes regarding pain management of labour in early or even before pregnancy, as women have voiced preferences about essential aspects of management of intrapartum care preconceptionally and early on in pregnancy. (Fontein-Kuipers et al., 2017)

The maternity care setting, environment and/or work culture shape the context and meaning of labour and labour pain management. (Fontein, 2010; Aune et al., 2021; Whitburn et al., 2017; Green and Baston, 2003) Caregivers’ beliefs about labour and birth, their beliefs in women's ability to give birth with or without pharmacological pain relief, affects care and information provision. (Leap et al., 2010; Aune et al., 2021) Therefore, to have conversations with pregnant women about comprehensibility, meaningfulness, and manageability of labour pain, requires reflection of care professionals of their own views on the salutogenic elements of intrapartum pain and pain management. (Schwartz et al., 2015; Magistretti et al., 2016) The National Institute for Health and Care Excellence (NICE 2017) points out that midwives must be aware of their own beliefs and values regarding pain during childbirth and that their preferences and interests concerning labour pain management should not influence the women's intention or preference. (Lindholm, 2015) When the woman has a trusting relationship with a known care provider, professionals should be aware whether women voice an intention to elicit support, empathy, and companionship of that professional. (Bastian et al., 2014) Moreover, traditionally, the relationship between maternity care professionals and women is hierarchical, something midwives and obstetricians must be aware of and not misuse this. (Lindholm, 2015) Beliefs of intrapartum pain management, workplace practices as well as relationships with women should therefore be part of reflective practice and education. (Fontein-Kuipers et al., 2018)

Although on average the LPRAQ-p scores in this study were low, our regression model shows that when women perceive their pregnancy as having a big impact on their body, their intention for pain relief during labour increases. We had no information why the predominantly healthy pregnant women in our study perceived pregnancy as a big impact. However, approximately 50% of pregnant women experience pain and discomfort during pregnancy, being accepted as normal and to be expected during pregnancy, receiving little intervention. (Greenwood and Stainton, 2001) Additionally, women can perceive their pregnant body to be out of their control and as transgressing the socially constructed ideal body and beauty image and attractiveness, including weight gain, changing body shape, and feeling uncomfortable during activities and daily functioning. (Hodgkinson et al., 2014) Nowadays women can create intensive idealistic images of pregnancy and motherhood, which might be difficult to keep up with while being pregnant and making coping with the physical aspect of pregnancy difficult. (Beeck van et al., 2019; Guardino and Dunkel Schetter, 2014; Loyal et al., 2021) The regression model also shows that when women are convinced that when they use pain relief, they will feel more self-confident during labour, their intention for pain relief increases. Maternal confidence reduces pain perception during labour while low self-confidence can lead to a sense of helplessness in relation to pain and to the ability to cope. (Whitburn et al., 2014; Sun et al., 2010) With pain relief there seems to be no need for coping with helplessness, as there is no helplessness felt. (Kiehl and White, 2003) Our observations indicate that discussing challenges in life such as pregnancy, birthing a child and labour-related pain are needed to support mothers in understanding and managing pain during labour as well as supporting women antenatally in building capacity and developing childbirth coping strategies. (NICE 2017; Lindström et al., 2017) Future research is needed to investigate who, when and how antenatal education and information of intrapartum pain management is presented and how this effects the intention for intrapartum pain management.

Our regression model showed that when women feel that pain relief is something they do for themselves and not for their partner, their intention for pain relief increases. Additionally, mean LPRAQ-p scores whether the partner, family, and friends are of influence in the decision-making process indicate that the women don't believe others have an influence on their decisions regarding pain relief. It seems to be the woman's decision, and her decision only - allowing her control over this aspect of birth. (Thomson et al., 2019; Skowronski, 2015; Green and Baston, 2003) This finding contradicts with all the recommendations to involve the partner during pregnancy and birth as this benefits the outcomes of pregnancy and birth as well with partners’ need to be involved and informed. (Bäckström and Hertfelt Wahn, 2011) Our finding also contradicts with women's wishes to have an involved, accessible, engaged, and responsible partner during pregnancy and birth. (Alio et al., 2013) Although most of the women in our study were in a relationship, we were unaware of the nature or quality of the couple's relationship or of women's emancipatory viewpoints about choice and control regarding pain management. It would be of interest to investigate the phenomenon with a qualitative research method. Additionally, we observed that, comparable with previous studies, women with antenatal fear of childbirth are more likely to prefer the use of pain relief during labour. (Logtenberg et al., 2018; Saisto et al., 2001; Fontein-Kuipers and Jomeen, 2019) This emphasizes the importance to address women's antenatal worries and fears in helping women to feel confident to openly and honest discuss their feelings and their related choices for pain relief. To aid the discussion, care professionals might benefit from understanding the importance of discussing birth-related fear throughout pregnancy. (Haines et al., 2012)

## 11 Limitations

There are some limitations to be mentioned. Because we did not follow up the women in our sample, we have no information about their actual intrapartum pain management. If discrepancies between intention and actual uptake occurred, we lack information whether the underlying factors had anything to do with the predicting factors in our regression model. As we questioned pregnant women, with labour still ahead, it can be assumed that the process of labour and/or coping with labour is an important contributing factor to the change between intention and the actual uptake of pain relief – not included in our study. Although our regression model predicted a large percentage of the variance of intention to pain relief during labour, (Cohen, 1988) our model is likely to be inconclusive and further research into underlying assumptions of women or other determinants is warranted. A longitudinal cohort study comparing women's levels of intention and actual pain management, including antenatal and intrapartum information and organisation of care, can be recommended.(Soliday et al., 2013, Lindholm, 2015) Due to the self-selective nature of our recruitment, it is plausible that women who completed the survey had a particular interest in the topic, causing bias. Self-selection, via social media platforms, might have led to sampling bias, although Facebook© can be a useful tool to recruit a specific target population. (Whitaker et al., 2017) We asked the participants about their pain relief intentions but only explicated epidural analgesia. Therefore, we did not know which other pharmacological pain relief women referred to and this could also have been interpreted as non-pharmacological pain relief methods. Pain relief counselling in Dutch maternity services, takes place at approximately 34 weeks’ gestation.(FMS 2020) We do not know how well-informed women in this study were regarding intrapartum pain management and if or how this affected their intention levels. Our sample consisted of predominantly of healthy women with uncomplicated pregnancies and included more parous than nulliparous women, possibly affecting the high proportion of low levels of intentions for pain relief.(Seijmonsbergen-Schermers et al., 2020) Our findings are therefore representative for low-risk women. For futures research, it can be recommended to recruit more high-risk women to avoid selection bias. The sample also included predominantly women with a Dutch background, in a relationship, with a job, and with high levels of education, also affecting the generalizability of the findings to women with other backgrounds or characteristics.

## 12 Conclusion

The maternity care professional who supports the woman during labour, the woman's attitude regarding the impact of pregnancy and her self-confidence in coping with pain during labour, and the nearly non-existent role of the woman's partner in the woman's intention for intrapartum pain management explained the differences of pregnant women's level of intention. The findings are mostly applicable to healthy pregnant women with uncomplicated pregnancies and as overall labour pain relief attitude scores were low, implies that findings are applicable to women with higher pain relief intentions. These key findings underlie the importance of further explorations in this area to determine whether these aspects worth paying attention to during the antenatal period to support low-risk women in pursuing their preferred pain management, but also for future research. The findings of this study inform the development and refinement of interventions to support women in their intentions for their intended management of pain during labour and might contribute to minimizing the pain management action-intention gap to prevent women's potential negative emotions and birth experiences.

## 13 Ethical approval

The Research and Ethics Committee of the Antwerp University Hospital approved the study protocol (Reference nr. B300201942200).

## 14 Funding

This research did not receive any specific grant from funding agencies in the public, commercial, or not-for-profit sectors.

## Declaration of Competing Interest

All authors disclose no actual or potential conflict of interest including any financial, personal or other relationships with other people or organizations within three years of beginning the submitted work that could inappropriately influence, or be perceived to influence, this work.
